# BF066, a Novel Dual Target Antiplatelet Agent without Significant Bleeding

**DOI:** 10.1371/journal.pone.0040451

**Published:** 2012-07-16

**Authors:** ChangE Pan, Xunbin Wei, Jianqin Ye, Guangda Liu, Si Zhang, Yan Zhang, Hongguang Du, Zhongren Ding

**Affiliations:** 1 School of Life Science, Fudan University, Shanghai, China; 2 Institutes of Biomedical Sciences, Fudan University, Shanghai, China; 3 Med-X Research Institute and School of Biomedical Engineering, Shanghai Jiao Tong University, Shanghai, China; 4 Key Laboratory of Molecular Medicine, Ministry of Education, and Department of Biochemistry and Molecular Biology, Fudan University Shanghai Medical College, Shanghai, China; 5 College of Science, Beijing University of Chemical Technology, Chaoyang District, Beijing, China; Temple University, United States of America

## Abstract

In this study, we report BF066, a novel adenine derivative, inhibits platelet activation and thrombosis via the adenosine receptor (A_2A_) activation and phosphodiesterase (PDE) inhibition. BF066 inhibits platelet aggregation and ATP releasing induced by multiple platelet agonists in a dose-dependent manner. The inhibition of BF066 on ADP-induced aggregation is potentiated by adenosine and can be dramatically antagonized by the A_2A_ antagonist SCH58261. BF066 also inhibits the PDE activity and platelet spreading on fibrinogen. In FeCl_3_-injured mouse mesenteric arterial thrombosis model, BF066 prevents thrombus formation effectively, similar to clopidogrel. Intriguingly, at dose achieving similar antithrombotic effect compared to clopidogrel, BF066 does not increase bleeding significantly. Taken together, these results suggest that BF066 may be an effective and safe antiplatelet agent targeting both PDE and A_2A_. Considering the successful use of combined antiplatelet therapy, BF066 may be further developed as a novel dual target antiplatelet agent.

## Introduction

Arterial thrombotic diseases, such as coronary heart disease and stroke, are the leading cause of morbidity and mortality worldwide. Platelet activation plays an essential role in the initiation and development of these arteriothrombotic diseases [1]–[3]. Accordingly, antiplatelet therapy has been established as a cornerstone in the management of arterial thrombotic diseases.

Many antiplatelet agents such as aspirin, a cyclooxygenase inhibitor, clopidogrel and prasugrel, thienopyridine class of the P2Y_12_ receptor antagonists, fibrinogen receptor antagonists, and cilostazol, a phosphodiesterase (PDE) inhibitor have been reported to be beneficial in patients with coronary heart disease, stroke and peripheral arterial disease [4]–[6]. Despite the proven benefits of currently available antiplatelet agents, there are still recurrent ischemic events; morbidity and mortality are still high [7]. This is because all the current available antiplatelet agents only target one signal pathway, and most of them inhibit platelet activation moderately and variably, especially for aspirin and clopidogrel. By blocking the final common pathway of platelet activation, fibrinogen receptor antagonists are very effective. However, their severe bleeding risk has limited them only for emergency use. Therefore, there is much room for further improvement of antiplatelet treatment and development of novel antiplatelet agents with increased efficacy and safety profile.

To achieve better clinical outcome with improved antithrombotic efficacy and safety, dual antiplatelet therapy with aspirin plus clopidogrel is widely used, while triple antiplatelet therapy (clopidogrel plus aspirin plus cilostazol) is under intensive evaluation [8], [9]. Clinical studies have confirmed that combination therapy results in enhanced antithrombotic efficacy without increasing bleeding risk [10], indicating that antiplatelet agents targeting multiple platelet activation pathways may be a promising strategy to develop more effective and safer antiplatelet agents.

**Figure 1:**
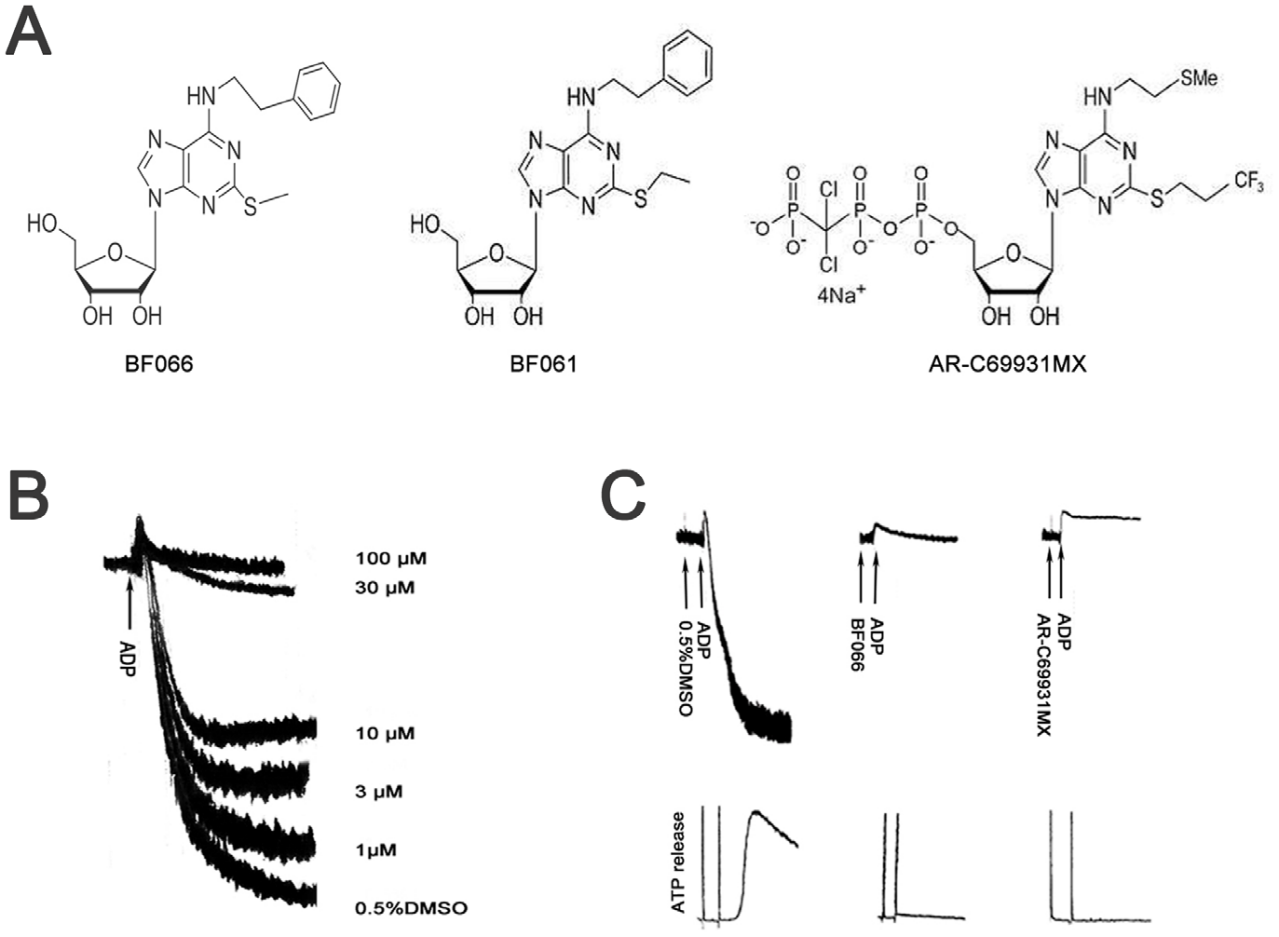
BF066 inhibited ADP-induced platelet activation. A) The structures of BF066, BF061 and AR-C69931MX. B) BF066 concentration-dependently inhibited ADP (10 µM) induced platelet aggregation in aspirin-treated human washed platelets. C) BF066 inhibited ADP-induced platelet aggregation and ATP release in non-aspirin treated human washed platelets. Platelets were pre-incubated with DMSO, BF066 (100 µM) or AR-C69931MX (100 nM) followed by stimulation with ADP (10 µM). BF066 (100 µM) pretreatment substantially inhibited platelet aggregation and abolished ATP release induced by ADP. Tracings shown are representatives of at least three experiments using platelets from different donors. DMSO was used as a vehicle control.

Previously, we reported a dual antiplatelet drug BF0801 targeting P2Y_12_ and PDE [11]. In order to improve the antiplatelet activity, we have modified BF0801, and obtained some novel chemicals. Among them, BF061 and BF066 (2-methylthio-6-phenethylaminoadenosine) (Figure 1) present the highest stability and solubility in water. BF061 has been reported to work on P2Y_12_ and PDE with an improved concentration [12]. In this study, we have investigated the antiplatelet and antithrombotic effects of BF066, and found out that it inhibits platelet activation and thrombosis via the adenosine receptor (A_2A_) activation and PDE inhibition without significant bleeding.

## Materials and Methods

### Regents and Chemicals

BF066 was synthesized by Institute of Materia Medica, Beijing University of Chemical Technology (Beijing, China). ADP was purchased from Chrono-Log Corp. (Havertown, PA, USA). Apyrase grade VII, adenosine, SCH58261, human fibrinogen, 3-isobutyl-1-methylxanthine (IBMX), 3, 5-cyclic adenosine monophosphate (cAMP), acetylsalicylic acid (aspirin), and calcein acetoxymethyl ester were purchased from Sigma-Aldrich (St Louis, MO, USA). AR-C69931MX was a gift from AstraZeneca (Loughborough, United Kingdom). Clopidogrel was from Sanofi-Aventis (Hangzhou, China). All other reagents were reagent grade. Deionized water was used throughout the experiments.

### Animals

The C57BL/6 mice used in this study were 8–15 weeks old unless otherwise stated. Animal procedures were carried out in accordance with institutional guidelines after Fudan University Animal Care and Use Committee approved the study protocol.

### Preparation of Human Platelet Rich Plasma and Washed Platelets

All experiments using human subjects were performed in accordance with the Declaration of Helsinki and approved by the Institutional Review Board Fudan University. Only healthy volunteers without taking aspirin or other nonsteroidal anti-inflammatory drugs for at least 14 days were recruited and written informed consent was obtained before blood collection. Thirty six mL blood was drawn into tubes containing 6 mL ACD (85 mM sodium citrate, 71.38 mM citric acid, and 27.78 mM glucose) solution. The blood was centrifuged at 300×g for 20 minutes to generate platelet rich plasma (PRP). If indicated, PRP was incubated with 1 mM aspirin for 30 minutes at 37°C. The PRP was then centrifuged at 900×g for 10 minutes to pellet the platelets. The resulting platelet pellet was resuspended in Tyrode’s buffer (138 mM NaCl, 2.7 mM KCl, 2 mM MgCl_2_, 0.42 mM NaH_2_PO_4_, 5 mM glucose, 10 mM HEPES, 0.2% bovine serum albumin, and 0.02 unit/ml apyrase; pH 7.4) to obtain washed platelets. Platelet number was adjusted to 2.5×10^8^ platelets/ml using Tyrode’s buffer [11], [12].

**Figure 2:**
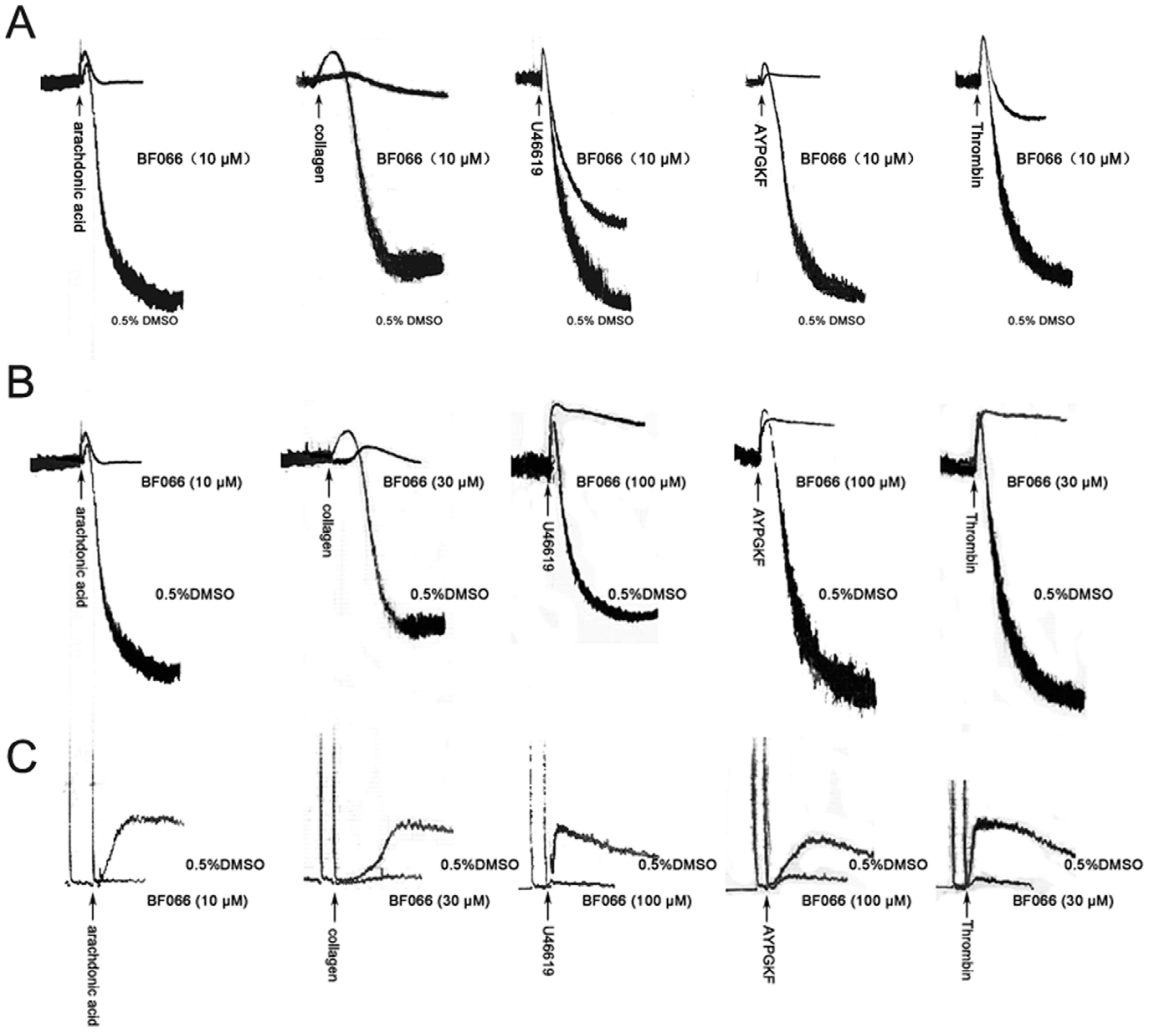
BF066 inhibited platelet activation induced by arachidonic acid, collagen, U46619, AYPGKF, and thrombin. A & B) BF066 (10–100 µM) abolished platelet aggregation in non-aspirin treated human washed platelets induced by 0.5 mM arachidonic acid, 2 ng/ml collagen, 1 µM U46619, 100 µM AYPGKF or 0.5 U/ml thrombin. C) Simultaneously recorded ATP release was inhibited by BF066. Tracings shown are representative of at least three experiments using platelets from different donors. DMSO was used as vehicle control.

### Preparation of Mouse Platelets

Blood was collected from the abdominal aorta of pentobarbital sodium anesthetised mice into syringes containing 100 µl/ml Whites anticoagulant (49.4 g/L sodium citrate, 24.6 g/L glucose), pH 6.4, 0.1 µg/ml PGE1, and 1 U/ml apyrase. Washed platelets were prepared and resuspended in Tyrode’s buffer at a final concentration of approximately 10^6^ platelets/µl as described previously [11], [13]. C57BL/6 mice (Animal Center of Fudan University) were anesthetized by intraperitoneal injection 10% chloral hydrate (400 mg/kg). At time zero, BF066 (29 mg/kg) or normal saline was injected into the tail vein. Blood (6.3 ml) was drawn into tubes containing 0.7 ml 3.8% sodium citrate 5 minutes (min) later by cannulation of common carotid artery and immediately centrifuged (300×g for 10 min) at room temperature to obtain PRP. Platelet number was adjusted to 5×10^8^/ml using Tyrode’s buffer.

### Measurement of Platelet Aggregation and Platelet Secretion

Aggregation of human washed platelets in response to agonist or antagonists was analyzed using lumi-aggregometer (Model 400VS, Chrono-Log, Haverston, PA, USA) under stirring conditions (900 rpm) at 37°C as reported before [11], [12], [14], [15]. In some experiments, platelet secretion was monitored by measuring ATP release using CHRONO-LUME reagent in parallel with aggregation [11].

### Preparation of cAMP-dependent Phosphodiesterase (cAMP-PDE) Extract from Human Platelets

Human blood 36 ml drawn into tubes containing 6 ml ACD solution was centrifuged at 300 g for 20 minutes to generate PRP. Platelets were further pelleted by centrifugation at 900 g for 10 minutes and were homogenized in 1 ml ice-cold 30 mM PBS containing 0.1% Trion X-100 using ultrasonic homogenizer (10 times for 5 seconds each at an interval of 5s, 50 Hz; Model UR-200P, Tokyo, Japan). After centrifugation (12,000×g for 20 min at 4°C using an Eppendorf Centrifuge 5415 R, Hamburg, Germany), the supernatant was collected and stored at −80°C until use or subjected to PDE activity assay.

**Figure 3:**
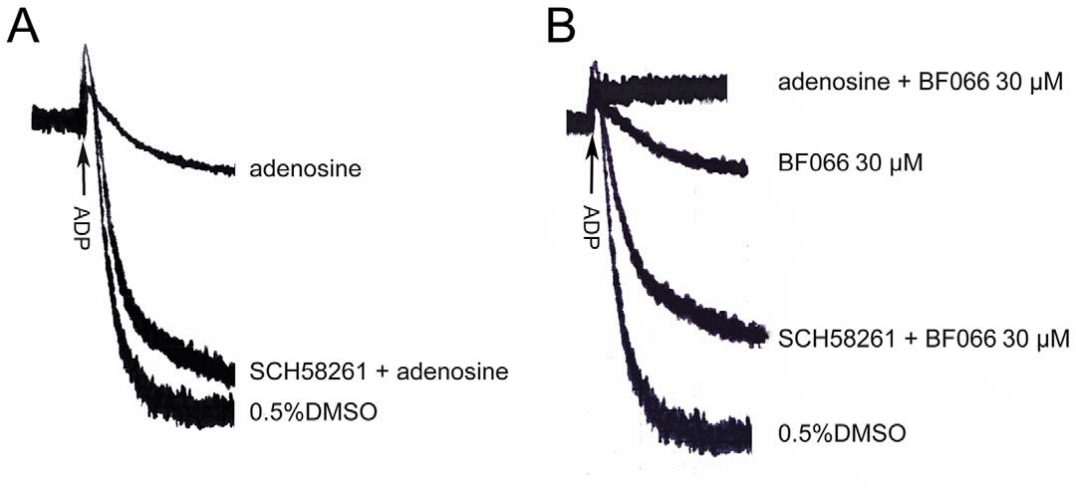
SCH58261 partially antagonized the inhibitory effect of BF066 on ADP-induced platelet aggregation. A) Adenosine (10 µM) inhibited platelet aggregation induced by ADP (10 µM) in aspirin-treated human washed platelets. B) The inhibition of BF066 (30 µM) on ADP-induced platelet aggregation was partially antagonized by 10 µM SCH58261. Tracings shown are representatives of at least three experiments using platelets from different donors. DMSO was used as vehicle control.

### Assay for cAMP-PDE Activity in Platelets

The assay for cAMP-PDE activity was performed using HPLC. Briefly, DMSO vehicle, IBMX 100 µM or different concentrations of BF066 were added to 200 µl assay buffer (137 mM NaCl, 2.7 mM KCl, 8.8 mM Na_2_HPO_4_, 1.5 mM KH_2_PO_4_, 1 mM CaCl_2_, 1 mM MgCl_2_, 10 µM cAMP, pH 7.4). Reaction was initiated by the addition of 10 µl PDE extracts prepared from human platelets as described above. In inactivated enzyme reaction system PDE extracts were boiled for 3 minutes before added. After incubation at 37°C for 30 minutes, the reaction was stopped by boiling the mixture for 3 minutes. The reaction mixture was cooled on ice, followed by centrifugation at 12,000×g for 30 minutes at 4°C. The cAMP in the supernatant was analyzed by HPLC (Kromasil 4.6×150 mm, Eka-chemicals, Bohns, Sweden). The amount of cAMP was determined using a standard curve of cAMP. The converted cAMP was measured as residual cAMP amount in inactivated enzyme reaction system minus residual cAMP amount in enzyme reaction system treated by different agent. The inhibition of PDE activity was calculated using the following formula: % inhibition of PDE activity = (1 - converted cAMP in enzyme reaction system treated by different agent/converted cAMP in untreated enzyme reaction system) ×100%.

**Figure 4:**
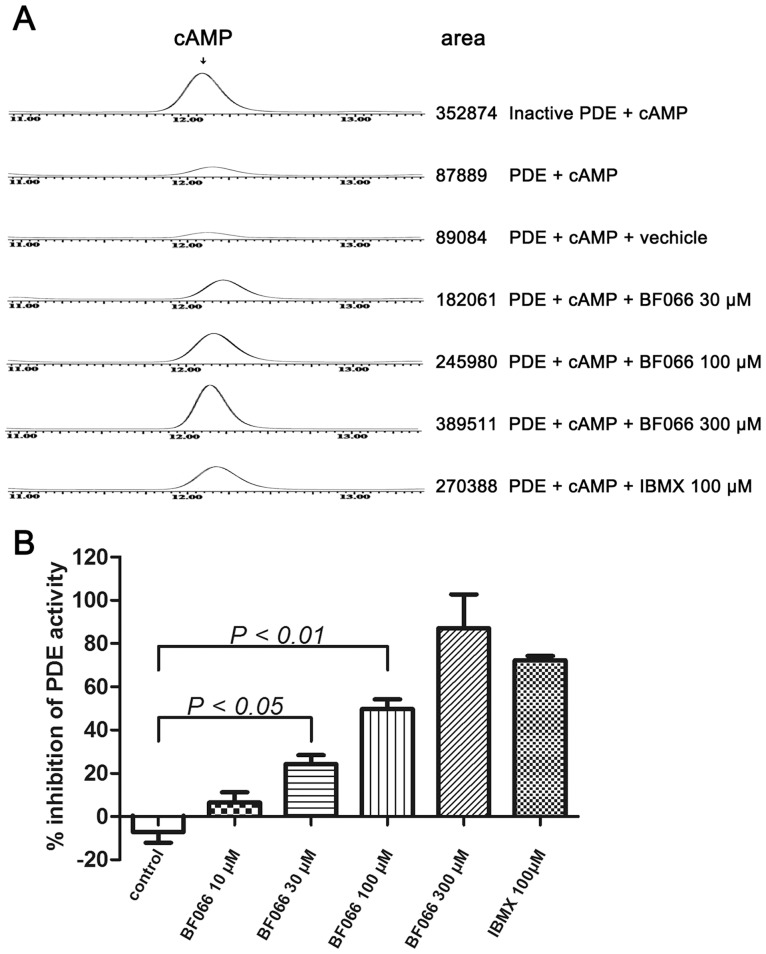
BF066 inhibits cAMP-phosphodiesterase activity in human platelet extracts. BF066 concentration-dependently inhibited activity of PDE extracted from human platelets. A) Representative HPLC tracings of residual cAMP in the presence of PDE and various inhibitors. B) BF066 concentration-dependently inhibited activity of PDE extracted from human platelets. Data were shown as mean ± SEM representing four separate experiments.

### Intravital Microscopy of FeCl_3_-induced Thrombosis in Mouse Mesenteric Arteriole

Intravital microscopy was carried out according to the method reported with minor modification [12], [16], [17]. Mouse platelets were labeled fluorescently by incubating with calcein acetoxymethyl ester (0.25 µg/ml) in PIPES buffer (25 mM PIPES, 137 mM NaCl, 4 mM KCl, 0.1% dextrose, pH 7.0) for 15 min at room temperature. After a bolus injection of BF066 (29 mg/kg, in 200 µl PIPES buffer) or same volume vehicle via tail vein, 6–8 weeks old C57BL/6 mice were injected with labeled platelets (4−5×10^9^/kg) in 200 µl PIPES buffer via lateral tail vein. The mice were anesthetized immediately with pentobarbital and the mesentery was exteriorized gently through a midline abdominal incision. Mesenteric arterioles of 70–100 µm diameters were chosen to expose within 30 min of injection of labeled platelets and visualized with a Leica DM 5500 Q microscope (objective: 10×) connected to Leica TCS SPE confocal system (Leica Microsystems, Mannheim, Germany). Arterioles were filmed for 1 min. Whatman paper (1 mm×2 mm) saturated with FeCl_3_ solution (10%) was applied topically for 1 min, and video recording was resumed for another 20 min.

### Platelet Spreading on Immobilized Fibrinogen

Human washed platelets were transferred to the slides to adhere for 90 minutes at 37°C, which were pre-coated overnight with either human fibrinogen (20 µg/ml) in 0.1 M of NaHCO_3_, pH 8.3 at 4°C [18]. After washing with phosphate-buffered saline (PBS), the adherent platelets were fixed with 1% paraformaldehyde, then followed by an incubation with splitting solution (0.1 M Tris, 0.01 M EGTA, 0.5 M NaCl, 5 mM MgCl_2_, 0.1% Triton X-100, 0.5 mM leuptin, 1 mM PMSF, 0.1 mM E64, pH 7.4) for 10 minutes at room temperature. Platelets were stained with FITC-phalloidin at room temperature for 30 minutes, followed by three times of washing with PBS [19]. The adherent platelets were examined by confocal microscopy using a Leica SPE confocal microscope with a 40× objective. The images were processed with Leica LAS AF Lite software [12].

**Figure 5:**
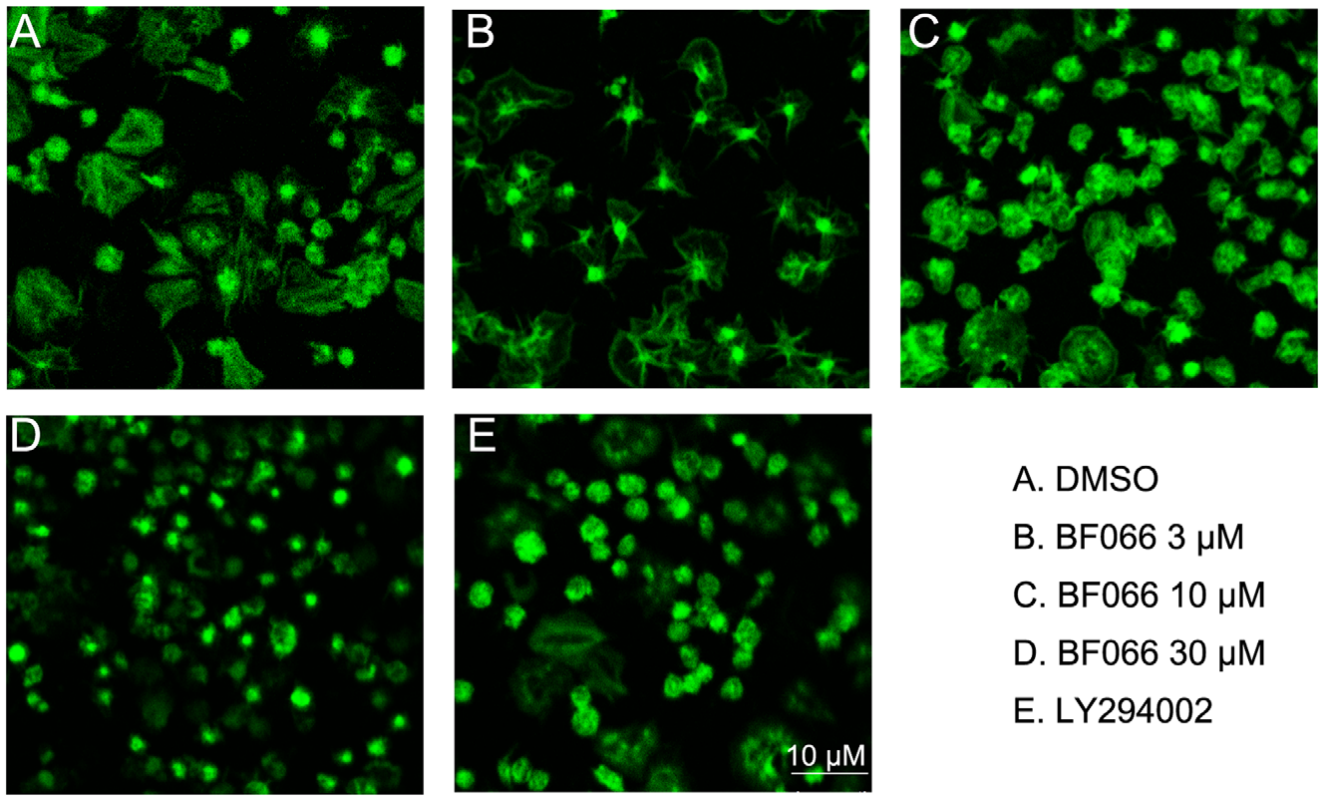
BF066 inhibits platelet spreading on immobilized fibrinogen. Human washed platelets were seeded on fibrinogen-coated cover slips in the presence of BF066 at different concentrations, PI3-kinase inhibitor LY294002 (100 nM) or DMSO. Platelets were stained with FITC-phalloidin and observed under a 40× objective with magnification of 5.

### Bleeding Assay

C57BL/6 mice anesthetized with benzobarbital (100 mg/kg, i.p.) were placed prone on a warming pad from which the tail protruded. The distal 5 mm of the tail was transected and immersed for 10 min in 1 ml saline warmed to 37°C. Absorbance of saline at 560 nm was measured and blood loss was calculated using a standard curve produced from known volumes of mouse blood. Same amount of BF066 (29 mg/kg, i.v., once) and clopidogrel (30 mg/kg, p.o., once daily for 2 days) as in thrombosis model were given [17], [20].

### Statistical Analysis

All data are expressed as mean ± SD. Differences between the groups were analyzed by one-way analysis of variance (ANOVA) followed by a Newman-Keuls test using GraphPad Prism version 5.0 unless otherwise stated. *P* values less than 0.05 were considered statistically significant.

**Figure 6:**
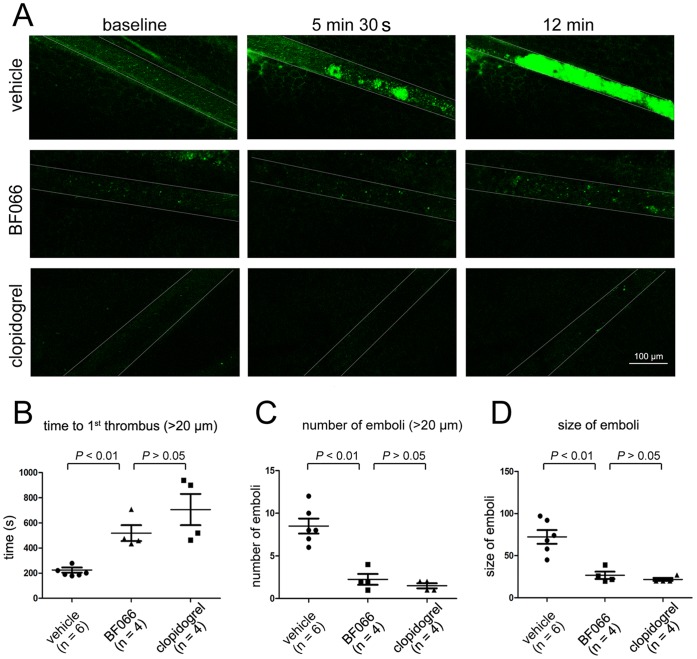
BF066 inhibits FeCl_3_-induced thrombus formation in mesenteric arterioles of C57BL/6 mice. A) Representative images of thrombus formation at baseline, 5.5 and 12 min after 10% FeCl3-injured vascular injury in mouse mesenteric arterioles. Blood flow is from right to left. Arterioles with a diameter of 70–100 µm were visualized in mesentery of live mice. Video S1 and video S2 show the effect of BF066 and clopidogrel on thrombus growth are available online. B) The time to form first thrombus (>20 µm), number of emboli (>20 µm) lasting over 2 min, size of emboli over 2 min starting from the time of the first thrombus [33] in mesenteric arterioles of untreated and treated mice were determined. Data are expressed as mean ± SEM.

## Results

### Inhibitory Effects of BF066 on Platelet Aggregation and ATP Release Induced by ADP

ADP is very important in platelet activation and thrombosis. BF066 is structurally similar to BF061 and AR-C69931MX (Figure 1A), both target P2Y_12_ receptor and inhibit platelet activation induced by ADP. Therefore, we first studied whether BF066 inhibits platelet activation induced by ADP. As shown in Figure 1B, BF066 dose-dependently inhibited platelet aggregation elicited by 10 µM ADP in aspirin-treated human washed platelets with an IC_50_ of 5.23 µM (Figure 1B). In non-aspirin treated human washed platelets, 100 µM BF066 abolished platelet aggregation and ATP release induced by 10 µM ADP (Figure 1C), with similar efficacy to AR-C69931MX, an antiplatelet agent currently under clinical trial.

### Inhibitory Effects of BF066 on Platelet Aggregation and ATP Release Induced by Arachidonic Acid, Collagen, U46619, AYPGKF and Thrombin

Platelets are activated via a complex signal transduction cascade mediated by multiple agonists including ADP, thrombin, collagen and thromboxane. Hence, after showing excellent antiplatelet efficacy of BF066 using ADP as a stimulus, we further explored the antiplatelet effects of BF066 on platelet activation induced by arachidonic acid, collagen, U46619, AYPGKF and thrombin. As shown in Figure 2, in the range of 10–100 µM, BF066 also abolished or drastically inhibited platelet aggregation and ATP release in human washed platelets induced by arachidonic acid, collagen, U46619, AYPGKF and thrombin.

**Figure 7:**
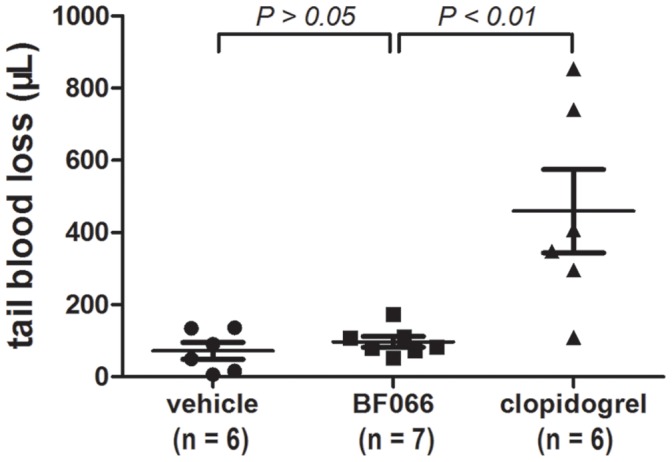
Effects of BF066 on bleeding. C57BL/6 mice anesthetized with pentobarbital (100 mg/kg) were placed prone on a warming pad from which the tails were protruded. The distal 5 mm of the tail was transected and immersed in 12 ml saline kept at 37°C for 10 min, blood loss was measured as described in Materials and methods. Same amount of BF066 (29 mg/kg, i.v., once) and (clopidogrel, 30 mg/kg, p.o., once daily for two days) as in thrombosis model were given. Each dot represents the blood loss volume measured in individual mouse, mean ± SEM is also shown.

### Effects of BF066 and Adenosine on ADP-induced Platelet Aggregation

Adenosine, a known Gs-coupled receptor agonist, activates platelet adenosine receptor (A_2A_), increases cAMP and inhibits platelet aggregation [21]. As shown in Figure 2A, adenosine inhibited ADP-induced platelet aggregation, which was reversed by SCH58261, a A_2A_ receptor antagonist [17]. Similarly, SCH58261 dramatically antagonized the inhibitory effect of BF066 on ADP-induced platelet aggregation, confirming that BF066 inhibits platelet activation via A_2A_ receptor activation (Figure 3B). Notably, the antagonizing effect of SCH58261 on the role of BF066 is weaker than that of adenosine, which suggests that BF066 may exert its antiplatelet role via an additional mechanism (Figure 3B). This is confirmed by our finding that adenosine further potentiated the inhibition of BF066 on ADP-induced platelet aggregation (Figure 3B).

### Inhibitory Effect of BF066 on the Activity of PDE Extracted from Human Platelets

BF066 has similar structure compared with BF061, which inhibits platelet activation via PDE inhibition and P2Y_12_ antagonism [12]. To explore another antiplatelet mechanism of BF066 in addition to A_2A_ receptor activation as implicated in Figure 3B, we extracted PDE from human platelets and investigated the effect of BF066 on PDE activity indicated by residual cAMP using HPLC. As shown in Figure 4, at concentrations ranging from 10 to 300 µM, BF066 inhibited the activity of platelet PDE extracts. The inhibition was concentration dependent, as the residual cAMP content increased in accordance with the concentration of BF066 (Figure 4). In addition, the inhibitory effect of BF066 on platelet PDE was comparable to that of IBMX at the same concentration.

### Inhibitory Effect of BF066 on Platelet Spreading on Immobilized Fibrinogen

Platelet aggregation is the result of fibrinogen binding to the activated integrin GPIIb/IIIa mediated by inside-out signal pathways. Upon GPIIb/IIIa binding to fibrinogen, it triggers outside-in signaling, causing platelet spreading and clot retraction which play a crucial role in thrombosis. After showing that BF066 inhibits platelet aggregation, dense granule release, we seek to explore whether it also influence platelet outside-in signaling. As shown in Figure 4, in the range of 3–30 µM, BF066 inhibited platelet spreading on immobilized fibrinogen in a dose-dependent manner (Figure 5). BF066 30 µM exhibited comparable inhibition on platelet spreading as LY294002, a PI3K inhibitor, which has been reported to inhibit platelet spreading.

### Inhibitory Effects of BF066 on FeCl_3_-induced Thrombus Formation in Mouse Mesenteric Arteriole

The dramatically inhibitory effects of BF066 on platelet aggregation, dense granule release and spreading on immobilized fibrinogen indicate that it may be effective to inhibit thrombus formation in vivo. To examine the in vivo antithrombotic activity of BF066, we evaluated the effects of BF066 on FeCl_3_-injured thrombus formation in mouse mesenteric arterioles in vivo using intravital microscopy. As shown in Figure 6A, in untreated mice, multiple thrombi more than 30 µm were observed in the mesenteric arteriole at 5.5 min. At 12 min the arteriole was totally blocked by a stable bulky thrombus (Figure 6A, see Video S1 available online), occlusion occurred in all untreated mice within 14 min after FeCl_3_ injury (occlusion time is 747±61 s, n = 6). In contrast, one bolus injection of BF066 (29 mg/kg, in 200 µl PIPES buffer) 30 min before FeCl_3_ injury prevented thrombus formation over 12 min after FeCl_3_ injury (Figure 6A). No occlusion occurred in all BF066-treated mice over 14 min after FeCl_3_ injury, similarly to the mice treated with clopidogrel (30 mg/kg, p.o., once daily for two days; Figure 6A, Video S2 and Video S3 available online).

Further analysis revealed that the time to form the first thrombus (>20 µm) was drastically prolonged in BF066-trated mice compared to the mice receiving the same volume of vehicle. We observed the first thrombus at 519±127 s in BF066-treated mice (n = 4), whereas the time is 222±40 s in the vehicle-treated mice (n = 6, *P*<0.01). The number of emboli (>20 µm) in 2 min and the size of the emboli in the 2 min from the time of the first thrombus [22] were all markedly decreased by BF066 (Figure 6B). Taken together, these data confirm the in vivo antithrombotic role of BF066.

Currently clopidogrel is the gold standard to evaluate the antithrombotic efficacy of novel antiplatelet drugs; therefore, we compared the antithrombotic role of BF061 with clopidogrel. Under our conditions, both BF066 (29 mg/kg, a single bolus i.v. injection) and clopidogrel (30 mg/kg, p.o., once daily for two days) similarly prevented FeCl_3_-induced thrombosis over 12 min (Figure 6A, also see Videos S2 and S3 available online), drastically prolonged thrombus formation time, decreased the number and size of emboli in the mesenteric artery of C57BL/6 mice (Figure 6B), exhibiting similar antithrombotic efficacy (Figure 6), especially when vessel occlusion is evaluated.

### Effects of BF066 on Bleeding

To test the possible bleeding side effect of BF066, we measured BF066-induced C57BL/6 mouse blood loss after tail snip at the same concentration (29 mg/kg, a single bolus i.v. injection) used for in vivo antithrombotic study. The blood loss induced by BF066 is 97±39 µl, which is similar to the vehicle (72±57 µl, *P*>0.05), and negligible compared to the blood loss of 459±282 µl induced by clopidogrel (30 mg/kg, p.o., once daily for two days; Figure 7).

## Discussion

Combined antiplatelet therapy has been widely used clinically with improved clinical outcome. In our pursuit to develop more effective and safer antiplatelet agent, we previously reported a dual target antiplatelet agent which blocks P2Y_12_ and PDE, which has similar antiplatelet efficacy and much less bleeding risk compared to clopidogrel [23]. In this study we report another dual target antiplatelet agent, BF066, which inhibits platelet activation by activating platelet adenine receptor A_2A_ and inhibiting PDE. Using FeCl_3_-injured mouse mesenteric arteriole thrombosis model, we have found out that BF066 has similar in vivo antithrombotic efficacy as clopidogrel. Notably, at the antithrombotic concentration used, BF066 did not incur bleeding significantly measured by tail snip. These findings indicate that BF066 is an effective and safe antiplatelet agent which should be further developed for clinical use.

Platelets are activated by different activators via complex signal pathways. Among the multiple signaling molecules regulating platelet activation and thrombosis, cAMP plays a crucial role in the pathway. Though cAMP decrease does not activate platelets, intracellular cAMP increase inhibits platelet aggregation [24]–[26]. The level of cAMP is dependent on its rate of synthesis by adenylate cyclase and its rate of hydrolysis by cAMP-phosphodiesterases (PDEs). Cilostazol, a PDE inhibitor which increases intraplatelet cAMP and therefore inhibits platelet aggregation, has been widely used in Asia and currently under clinical trial in western countries as an antiplatelet agent [27]–[29]. Adenosine, which activates Gs-coupled adenosine receptor A_2A_, increases intraplatelet cAMP by activating adenylate cyclase and inhibits platelet activation [30] (Figure 3A).

BF066 inhibited platelet activation elicited by a broad range of agonists (Figure 1 & 2). Therefore, we speculate that it may block a common signal pathway involved in platelet activation. Considering that BF066 is an adenosine derivative and the antiplatelet role of adenosine, we first explored its possible antiplatelet mechanism via activation A_2A_, the major subtype of adenosine receptors in platelets [30]. The dramatic antagonism of the inhibition of BF066 by SCH58261, a specific A_2A_ antagonist, confirms that A_2A_ activation mediates the antiplatelet effects of BF066 (Figure 3B). Interestingly, though structurally similar to BF066 [12], it seems that the antiplatelet effect of BF061 does not involve A_2A_ activation, because its inhibition on ADP-induced platelet aggregation is not affected by SCH58261 (Figure S2).

Compared to the nearly abolishment of SCH58261 on the antiplatelet role of adenosine, the weaker antagonism of SCH58261 on the effect of BF066 indicate an extra mechanism underlying the antiplatelet role of BF066 in addition to A_2A_ activation. The synergism between the BF066 and adenosine on the inhibition of platelet activation is correlated with the involvement of an additional antiplatelet mechanism of BF066 beyond adenosine receptor activation, which is proved to be PDE inhibition. We also measured the effects of BF066 on TXA_2_ production and found out that it did not affect platelet TXA_2_ production (data not shown). As the analogue of BF061, which targets both PDE and P2Y_12_ [12], whether BF066 also works as P2Y_12_ antagonist need to be further investigated. VASP phosphorylation decrease induced by ADP is generally accepted to reflect P2Y_12_ activation. Though BF066 does reverse the decrease of VASP phosphorylation induced by ADP, but this could be the results of PDE inhibition and A_2A_ activation (Figure S1). The robust VASP phosphorylation in the presence of BF066 is in line with its PDE inhibition and A_2A_ activation (Figure S1).

It is known that PKA is a strong inhibitor for platelet activation. Because BF066 inhibits platelet activation through activating adenosine receptor A_2A_ and inhibiting PDE, inhibition of platelet activation by BF066 may be mediated by the PKA pathway. Therefore, we tested whether PKA inhibitors could reverse the inhibitory effect of BF066 on platelet aggregation. We did not notice the effects of PKA inhibitors PKI (12–4) [31] and H-89 on the antiplatelet role of BF066 (Figure S3). Hence, BF066 may inhibit platelet activation via a PKA-independent manner. Consistent with our findings, Hayashi et al [32] also found that cAMP-elevating agents cilostamide, cilostazol and forskolin inhibit platelet activation in a PKA-independent manner.

All currently available antiplatelet drugs inevitably increase bleeding risk at the antithrombotic doses, which limits their use to achieve better antithrombotic effects by increasing doses. Compared with our previously reported dual antiplatelet agent BF061, which has similar antithrombotic efficacy with less bleeding compared to clopidogrel [23], BF066 reported in this study possesses similar antithrombotic efficacy without significant bleeding; this makes BF066 more promising as an antiplatelet agent for further development to prevent and treat arteriothrombotic diseases including coronary artery disease and stroke.

Platelet outside-in signal pathway is mainly involved in thrombosis rather than hemostasis of platelet function. Targeting platelet outside-in signal pathway represents a more attractive approach to develop effective and safe antiplatelet agents [18]. The profound inhibition of BF066 on platelet spreading may contribute to its negligible bleeding tendency while retaining excellent antithrombotic efficacy.

## Supporting Information

Figure S1 **PKA inhibitor PKI (14–22) inhibits forskolin-induced VASP phosphorylation in human platelets.** Human washed platelets were preincubated with PKA inhibitor PKI (14–22) (12 µM) or BF066 (30 µM) for 15 min or 2 min at 37°C, respectively, followed by stimulation with forskolin (20 µM) or ADP (10 µM) for 3.5 min. VASP phosphorylation was detected via Western blot. Data shown are representative of 2 experiments using platelets from different donors.
(TIFF)

Figure S2 **SCH58261 does not antagonize the inhibition of BF061 on aggregation induced by ADP.** A_2A_ receptor antagonist SCH58261 (10 µM) did not block the inhibitory role of BF061 (10 µM) on ADP-induced platelet aggregation in aspirin-treated human washed platelets. As a control, SCH58261 almost abolished the inhibition of adenosine (10 µM) on platelet aggregation induced by ADP. Tracings shown are representative of at least 2 experiments using platelets from different donors.
(TIFF)

Figure S3 **PKA inhibitors PKI (14–22) and H-89 do not reverse the inhibition of BF066 on ADP-induced platelet aggregation.** Human washed platelets were preincubated with vehicle or PKA inhibitors PKI (14–22) (12 µM) and H-89 (19 µM) at 37°C for 18 min and 8 min, respectively, followed by incubation with BF066 (30 µM) or vehicle for another 1 min. Platelet aggregation was induced by addition of ADP 10 µM. Tracings shown are representative of 3 experiments using platelets from different donors. DMSO was used as a vehicle control. The activity of PKI (14–22) was proved by its inhibition on forskolin-induced VASP phosphorylation as shown in Fig. S1.
(TIFF)

Video S1 **FeCl_3_–induced thrombosis in a mouse mesenteric arteriole.** Multiple thrombi more than 30 µm were observed in the mesenteric arteriole at 5.5 min. At 12 min the arteriole was totally blocked by a stable bulky thrombus, occlusion occurred in all untreated mice within 14 min after FeCl_3_ injury Platelets were labeled with calcein.
(WMV)

Video S2 **BF066 prevents FeCl_3_-induced thrombosis in a mouse mesenteric arteriole.** One bolus injection of BF066 (29 mg/kg, i.v.) 30 min before FeCl_3_ injury prevented thrombus formation in a C57BL/6 mouse mesenteric arteriole over 12 min. Platelets were labeled with calcein.
(WMV)

Video S3 **Clopidogrel prevents FeCl_3_-induced thrombosis in a mouse mesenteric arteriole.** Clopidogrel (30 mg/kg, p.o., once daily for 2 days) prevented FeCl_3_-induced thrombus formation in a C57BL/6 mouse mesenteric arteriole over 12 min. Platelets were labeled with calcein.
(WMV)
