# Supplementary figures and images for: BF066, a Novel Dual Target Antiplatelet Agent without Significant Bleeding
Source: PLoS One. 2012 Jul 16;7(7):e40451. doi: 10.1371/journal.pone.0040451 (PMC3398006; doi:10.1371/journal.pone.0040451)

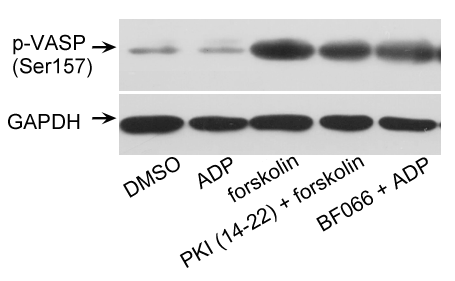

Supplement: Figure S1 — PKA inhibitor PKI (14–22) inhibits forskolin-induced VASP phosphorylation in human platelets. Human washed platelets were preincubated with PKA inhibitor PKI (14–22) (12 µM) or BF066 (30 µM) for 15 min or 2 min at 37°C, respectively, followed by stimulation with forskolin (20 µM) or ADP (10 µM) for 3.5 min. VASP phosphorylation was detected via Western blot. Data shown are representative of 2 experiments using platelets from different donors. (TIFF) [file pone.0040451.s001.tiff]

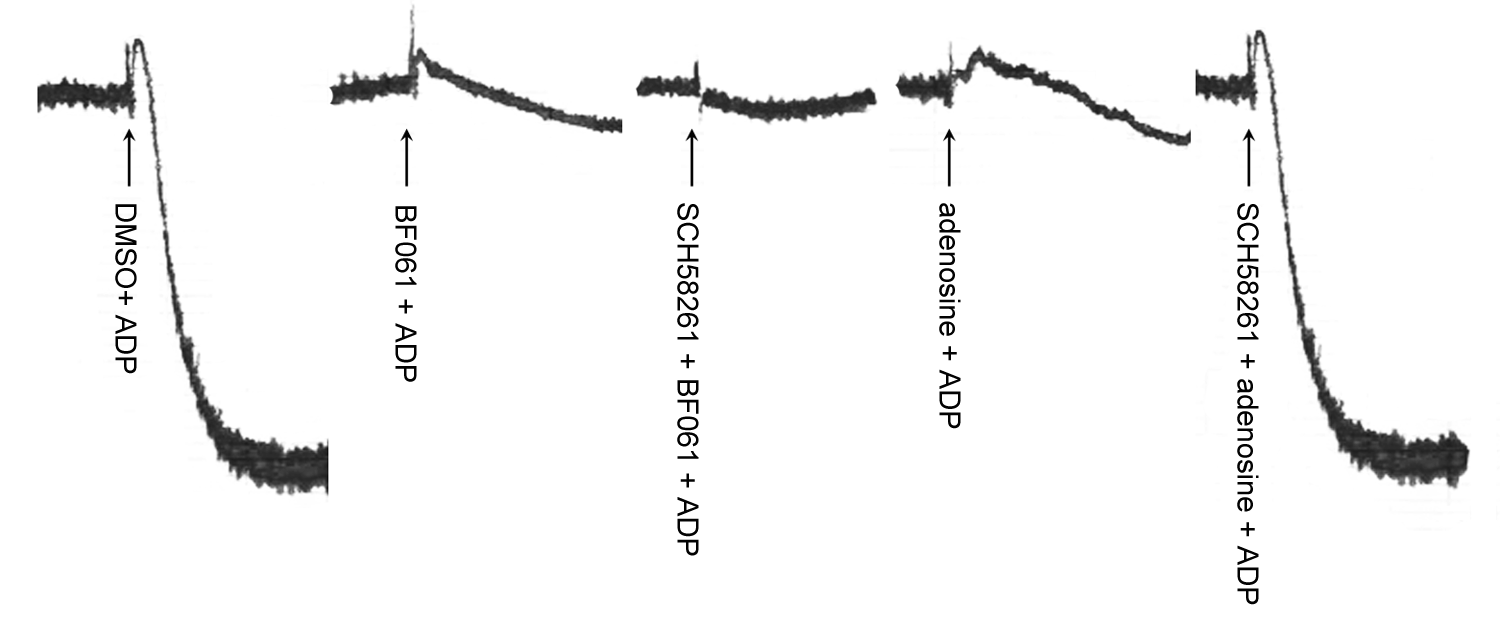

Supplement: Figure S2 — SCH58261 does not antagonize the inhibition of BF061 on aggregation induced by ADP. A2A receptor antagonist SCH58261 (10 µM) did not block the inhibitory role of BF061 (10 µM) on ADP-induced platelet aggregation in aspirin-treated human washed platelets. As a control, SCH58261 almost abolished the inhibition of adenosine (10 µM) on platelet aggregation induced by ADP. Tracings shown are representative of at least 2 experiments using platelets from different donors. (TIFF) [file pone.0040451.s002.tiff]

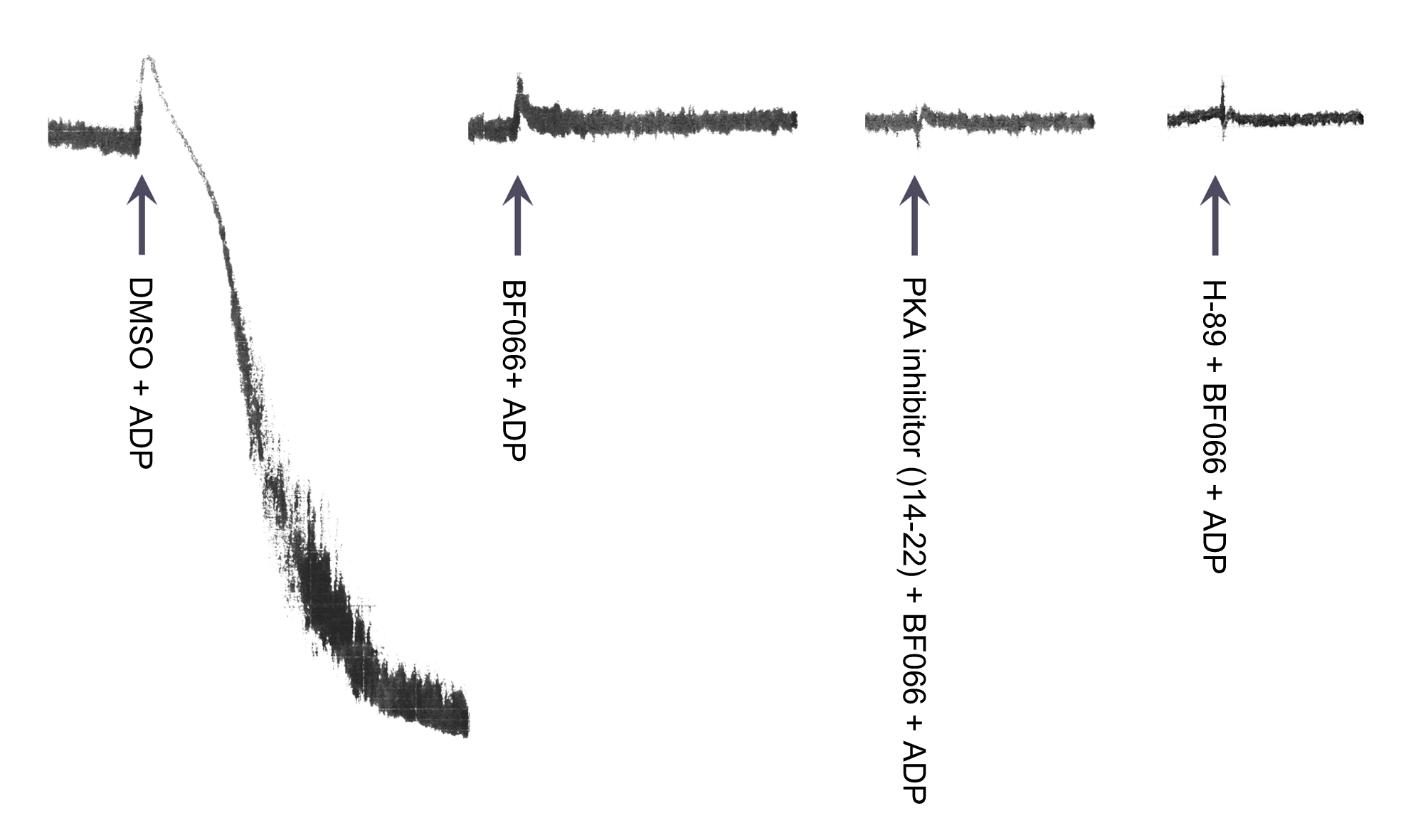

Supplement: Figure S3 — PKA inhibitors PKI (14–22) and H-89 do not reverse the inhibition of BF066 on ADP-induced platelet aggregation. Human washed platelets were preincubated with vehicle or PKA inhibitors PKI (14–22) (12 µM) and H-89 (19 µM) at 37°C for 18 min and 8 min, respectively, followed by incubation with BF066 (30 µM) or vehicle for another 1 min. Platelet aggregation was induced by addition of ADP 10 µM. Tracings shown are representative of 3 experiments using platelets from different donors. DMSO was used as a vehicle control. The activity of PKI (14–22) was proved by its inhibition on forskolin-induced VASP phosphorylation as shown in Fig. S1. (TIFF) [file pone.0040451.s003.tiff]
